# Antithetical roles of tau in hippocampal long-term synaptic depression in rats versus mice

**DOI:** 10.1186/s13041-026-01341-y

**Published:** 2026-10-23

**Authors:** Liam T. Ralph, Eric W. Salter, Patrick Tidball, Brian Park, Sun-Lim Choi, Lijia Zhang, Meng Tian, Ashish Kadia, Fuzi Jin, Clarrisa A. Bradley, Paul E. Fraser, Gerold Schmitt-Ulms, Marina Gertsenstein, Lauryl M. J. Nutter, John Georgiou, Graham L. Collingridge

**Affiliations:** 1 Lunenfeld-Tanenbaum Research Institute, Mount Sinai Hospital, Sinai Health System, Toronto, ON M5G 1X5 Canada; 2 Department of Physiology, University of Toronto, Toronto, ON M5S 1A8 Canada; 3 TANZ Centre for Research in Neurodegenerative Diseases, University of Toronto, Toronto, ON M5S 1A8 Canada; 4 Department of Neuroscience, Brown University, Providence, RI 02906 USA; 5 Neuroscience and Mental Health, Peter Gilgan Centre for Research and Learning, The Hospital for Sick Children, Toronto, ON M5G 1X8 Canada; 6 Department of Medical Biophysics, University of Toronto, Toronto, ON M5S 1A8 Canada; 7 Department of Laboratory Medicine and Pathobiology, University of Toronto, Toronto, ON M5S 1A8 Canada; 8 The Centre for Phenogenomics, Toronto, ON M5T 3H7 Canada; 9 Genetics and Genome Biology, The Hospital for Sick Children, Toronto, ON M5G 1X8 Canada

**Keywords:** Tau proteins, Long-term synaptic depression, Species specificity, Synaptic plasticity, Hippocampus, Synapse loss

## Abstract

Synaptic dysfunction is a primary feature of tauopathies, a group of major neurodegenerative diseases that includes Alzheimer’s disease. However, the normal synaptic functions of tau are poorly understood. Since rats provide advantages over mice for understanding human tau, such as expressing six tau isoforms and showing more robust signs of tau pathology, as well as offering a richer behavioural repertoire, we developed a tau knockout (*Mapt* KO) rat line. We primarily investigated the impact of tau elimination on long-term synaptic depression (LTD)—a form of synaptic weakening that tau mediates in mice. Low-frequency stimulation of hippocampal Schaffer collateral–CA1 synapses revealed a robust enhancement of LTD in *Mapt* KO rats relative to WT littermates. In contrast, in interleaved experiments, *Mapt* KO mice exhibited reduced LTD compared to WT littermates, in line with previous reports. Together, our findings demonstrate a species-dependent role for endogenous tau in regulating hippocampal LTD, promoting it in mice yet constraining it in rats. Therefore, the functions of tau are not necessarily conserved in rodents, underscoring the importance of animal model choice.

**Supplementary Information:**

The online version contains supplementary material available at https://doi.org/10.1186/s13041-026-01341-y.

Tau, a microtubule-associated protein crucial for the maintenance of neuronal cytoskeletal architecture, is disrupted in tauopathies such as Alzheimer’s disease (AD) [1]. Beyond its canonical pool, tau is also present at synapses [2] where it regulates key synaptic functions [1]. Since synaptic weakening and loss of synapses are early features of AD and other tauopathies [3], understanding the synaptic roles of tau is imperative for developing more efficacious therapeutics [4]. To date, the impact of tau deletion on synaptic function has primarily been investigated in microtubule-associated protein tau homozygous knockout (*Mapt*^*−*/*−*^; *Mapt* KO) mice, where tau deletion results in deficits in long-term synaptic depression (LTD) in juveniles [2].

While studies have focused on the effects of tau deletion in mice, the *Mapt* gene exhibits considerable evolutionary divergence [5]. In the central nervous system (CNS) of humans, tau exists as six isoforms, each with distinct physiological functions and neuropathological profiles [6]. Unlike mice, rats express the same number of tau isoforms as humans, suggesting that rats may provide a better model for understanding human tau [7, 8]. Rats also more closely mimic aspects of human physiology [9] and offer a richer behavioural repertoire than mice [10], enabling more sensitive assessment of tau-dependent behaviours, while being less prone to environmental stressors [11] that drive tau phosphorylation and pathology [12]. Furthermore, there is evidence to suggest that, compared to mouse models of AD, tau pathology in rat AD models more closely resembles that of human AD patients [13], further supporting rats as a better translational model.

Synaptic weakening is relevant to neurodegenerative diseases involving tau [1, 3], and previous work has shown inhibition of hippocampal LTD in *Mapt* KO mice [2]. However, whether tau plays a comparable role in synaptic function in rats is unknown. To address this, we developed a constitutive rat *Mapt* KO line (SD-*Mapt*^em1Tcp^) using CRISPR-Cas9 technology (Fig. 1A and Methods in Supplementary Materials). Sanger sequencing confirmed deletion of the fourth through fifth exon of the *Mapt* gene (Fig. S1A). Elimination of tau protein expression was confirmed by immunoblotting of whole-hippocampal homogenates from juvenile rats, using both an *N*-terminal monoclonal (Cat#: MABN2406, MilliporeSigma [14]) and a *C*-terminal polyclonal (Cat #: A1103, ABclonal [15]) antibody, directed against epitopes upstream and downstream, respectively, of the deleted region (Fig. 1B, C). Pups were born at expected Mendelian ratios from heterozygous breeding pairs (Fig. S1B), and *Mapt* KO pups exhibited no gross defects or alterations in body, brain and hippocampal mass compared to wild-type (*Mapt*^+/+^; WT) littermates (Fig. S1C–E).

**Fig. 1:**
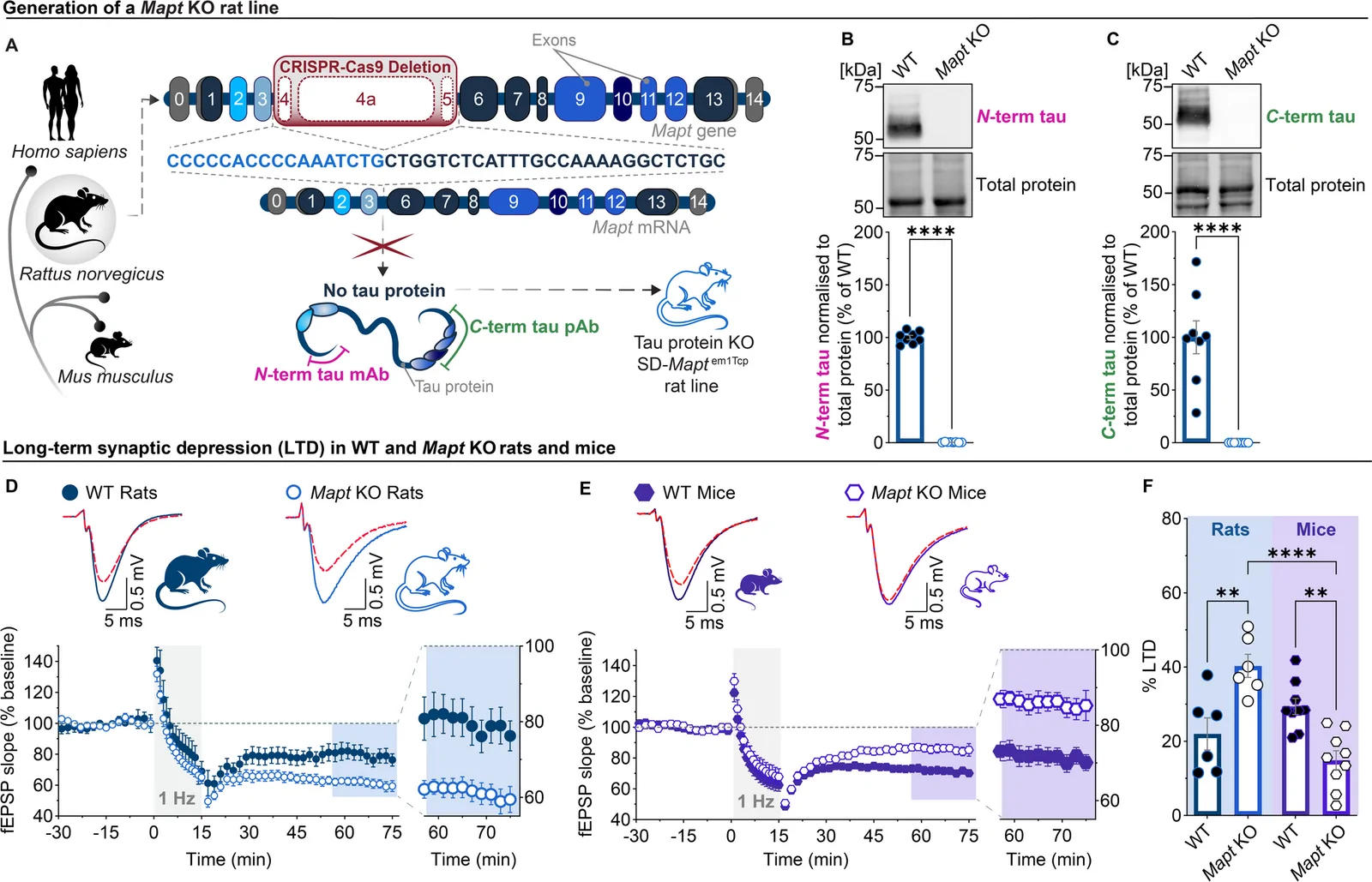
Opposing effects of *Mapt* deletion on LTD in male rats versus mice. **A** Schematics depicting the phylogenetic relationships between *Rattus norvegicus*, *Mus musculus*, and *Homo sapiens* (left), and the deleted region in the rat *Mapt* gene of the SD-*Mapt*^em1Tcp^ rat line (right). **B**, **C** Representative immunoblots (upper), total stain-free protein (middle), and quantification of tau protein levels (lower) in hippocampal homogenates from juvenile (P14–19) male *Mapt*^+*/*+^ (WT), and *Mapt*^*−/−*^ (KO) rats (*n* = 8 per group) using (**B**) an *N*-terminal monoclonal tau (cat #: MABN2406, Millipore Sigma) and (**C**) a *C*-terminal polyclonal tau (cat #: A1103, ABclonal) antibody, with their corresponding target regions shown on the tau protein schematic in panel A in pink (17 aa from the *N*-terminus) and green (aa 283–441 of 2N4R tau), respectively. Tau protein levels are normalised to total (stain-free) protein levels and expressed as a percentage of WT. Tau protein expression was absent in *Mapt* KO rats relative to WTs. Comparisons were made using unpaired two-tailed Student’s *t*-tests. **D**–**F** Time course of LFS-induced LTD (900 pulses delivered at 1 Hz) in WT (closed symbols) and *Mapt* KO (open symbols) male (**D**) rats (blue circles), and (**E**) mice (purple hexagons). Expanded regions of the time plots show the final 20 min of recordings. Representative traces show superimposed fEPSPs from the baseline period (solid blue/purple lines) and the end of the recording session (red dashed line). Levels of LTD (**F**) were calculated as the percent change in fEPSP slope from baseline in the last 6 min of recordings. Comparisons were made using two-way ANOVA followed by Šídák’s post hoc test. Detailed statistics are shown in Supplementary Tables 1 and 2. All data are presented as the mean ± S.E.M. **P < 0.01; ****P < 0.0001

To assess synaptic weakening in this model, we examined low-frequency stimulation (LFS)-induced LTD in the CA1 region of acute ex vivo hippocampal slices prepared from juvenile male *Mapt* KO rats and their male WT littermates. We also examined LFS-LTD in slices from juvenile male *Mapt* KO mice and their male WT littermates in interleaved experiments. Surprisingly, we found that the impact of tau deletion on LTD depended on rodent species (two-way ANOVA: species × genotype interaction, *F*_(1,26)_ = 30.5, *p* < 0.0001). Specifically, LTD was significantly enhanced in male *Mapt* KO rats (40.3 ± 3.1%, *n* = 6) relative to WT littermate controls (22.0 ± 4.3%, *n* = 6; *t*_(26)_ = 4.0, *p* = 0.002, Šídák’s post-hoc test, Fig. 1D, F). In contrast, LTD was reduced in male *Mapt* KO mice (14.9 ± 2.5%, *n* = 9) relative to WT littermates (29.4 ± 2.2%, *n* = 9; *t*_(26)_ = 3.9, *p* = 0.003, Fig. 1E, F), consistent with previous findings [2], and differed significantly from the augmented LTD obtained in male *Mapt* KO rats (*t*_(26)_ = 6.0, *p* < 0.0001). LTD levels between male WT mice and rats were similar (*t*_(26)_ = 1.8, *p* = 0.3). Together, these findings indicate that tau has an opposite role in LTD dependent on rodent species, facilitating LTD in mice and constraining LTD in rats.

Our interleaved experiments using male rats and mice confirm that this is a *bona fide* species difference rather than a difference in experimental conditions. The reason for this species difference remains unknown and could be attributable to several factors. One plausible explanation is that it arises from the function of a tau isoform that rats possess but mice do not (e.g., 1N3R/2N3R) since isoforms are known to have unique roles [6]. Extrinsic factors, such as differences in how rats and mice handle or respond to stress [11] for which tau has been shown to play a role in mice [12], could also contribute. A third plausible explanation relates to intrinsic factors, such as different expression levels of synaptic plasticity genes in dendrites including calcium/calmodulin-dependent protein kinase II (CaMKIIβ), neurogranin, and several synaptotagmin isoforms [9]. Thus, the underlying mechanisms of induction and/or expression of LTD may differ between these rodent species.

Our interest in studying tau deletion in rats stems from the evolutionary divergence in tau isoform expression [5]. Specifically, like humans, juvenile rats express all six CNS tau isoforms, and this persists into adulthood [7, 8]. In contrast, juvenile mice predominantly express the 0N3R isoform, which wanes with age, as well as the 0N4R tau isoform, which increases alongside 1N4R and 2N4R into adulthood [8], while expression of 1N3R and 2N3R isoforms is lacking [6]. These differences may be critical since different tau isoforms stabilise microtubules to different extents, have unique interaction partners related to synaptic function, and have distinct sub-cellular localisation patterns [6]. As such, rats are increasingly being used to study tauopathies. For instance, recent findings in male rats demonstrate that acute intracerebroventricular injection of oligomer-enriched tau facilitates LTD while fibril-derived soluble tau aggregates inhibit LTD [16]. Therefore, the generation of our *Mapt* KO rat line (SD-*Mapt*^em1Tcp^) offers a useful tool for studying the role of endogenous tau in AD and other neurodegenerative diseases.

Future experiments should consider (i) whether this species difference is also present in female rodents, given that sex differences in tau phosphorylation have been reported in rats [17], and (ii) how tau deletion impacts synaptic plasticity, learning and memory with age since the ratio of tau isoform expression [7, 8] and post-translational modifications [1] change across the lifespan. Indeed, tau deletion has been shown to not impact LTD induced by a stronger LFS protocol (2 Hz, 1800 pulses) in 6-month-old adult mice [18]. This may reflect the decline in LTD magnitude and a change of underling mechanism with age [19, 20]. Whether tau loss-of-function nonetheless reactivates LTD pathways in the context of tau pathology, and whether this differs between sexes or with age in rats, remains to be shown. In conclusion, the species difference we report here highlights the importance of selecting the most appropriate animal model for investigating the physiological and pathological functions of tau in relation to human health and disease.

## Supplementary Information

Additional file1 (PDF 169 KB)

Additional file2 (PDF 524 KB)

Additional file3 (DOCX 17 KB)

## Data Availability

All datasets are available from the corresponding authors upon reasonable request.

## Abbreviations

ACSF: Artificial cerebrospinal fluid
AD: Alzheimer’s disease
ANOVA: Analysis of variance
Cas9: CRISPR-associated protein 9
CNS: Central nervous system
CRISPR: Clustered regularly interspaced short palindromic repeats
fEPSP: Field excitatory postsynaptic potential
KO: Knockout
LFS: Low-frequency stimulation
LTD: Long-term synaptic depression
LTP: Long-term potentiation
*Mapt*: Microtubule-associated protein tau (gene)
WT: Wild-type
