# Supplementary material for: Antithetical roles of tau in hippocampal long-term synaptic depression in rats versus mice
Source: Mol Brain. 2026 Oct 23;19:76. doi: 10.1186/s13041-026-01341-y (PMC13692963; doi:10.1186/s13041-026-01341-y)
Supplement: Supplementary file 1 [file 13041_2026_1341_MOESM1_ESM.pdf]

Supplementary Materials

Methods

Creation of *Mapt* KO rat line

The constitutive **microtubule-associated protein tau homozygous knockout (*Mapt*$^{-/-}$; *Mapt* KO)** rat line (SD-*Mapt*^{em1Tcp}) was generated by delivering CRISPR-Cas9 reagents directly to Charles River Laboratories caesarean-derived Sprague Dawley (Crl:CD(SD)) rat zygotes, following established protocols (1). Sprague-Dawley (SD) rats were purchased from Charles River Laboratories (Montreal, QC) and maintained in the Canadian Council on Animal Care accredited animal facility at The Hospital for Sick Children (Toronto) in individually ventilated cages (Allentown) on a 12-h light/dark cycle (7 am on/7 pm off). Preparation of embryo donors and recipients, as well as embryo transfer surgeries were based on previously described procedures (2). Four guide RNAs were synthesized using EnGen sgRNA Synthesis Kit (Cat #: E3322, New England BioLabs) and were combined with 656 ng/µL of *Streptococcus pyogenes* Cas9 protein (cat #: 1074182, Integrated DNA Technologies). Electroporation was performed as previously described (1), but with modified electroporation parameters. Zygotes were briefly washed through Acidic Tyrode's solution (cat #: T1788, Sigma) and then Opti-MEM media (cat #: 31985062, ThermoFisher Scientific). The zygotes were then placed into 20 µL of Cas9 ribonucleoprotein mix and the whole volume was transferred to 1 mm-gap cuvette. Twelve square pulses at 30V with 3 ms pulse duration and 100 ms interval were applied using BioRad Gene Pulser XCell electroporator. The zygotes were retrieved from the cuvette with a Rainin pipettor and 20 µL tips followed by at least two rinses of 70 µL of Life Global media (LGGG-050, Cooper Surgical). Electroporated zygotes were implanted into pseudopregnant SD females. Founder progenies were screened for the desired polymerase chain reaction product (823 – 985

base pairs), and Sanger sequencing confirmed deletion of the fourth through fifth exons of the *Mapt* gene based on the NCBI reference sequence for full-length *Rattus norvegicus* CNS tau (XM_008768277.3; **Fig. S1A**). The deletion was predicted to introduce a premature stop codon, preventing protein expression of all tau isoforms. Tau protein knockout was confirmed in whole hippocampal lysates using an *N*-terminal specific monoclonal tau antibody (**Fig. 1B,C**).

Animals

Juvenile male (**postnatal day (P) 14–19**) **wild-type (*Mapt*$^{+/+}$; WT)** and *Mapt* KO rats (SD-*Mapt*^{em1Tcp}) and mice (B6.129X1-*Mapt*^{tm1Hnd}/J; (3)) were used in this study. **Heterozygous (*Mapt*$^{+/-}$; HET)** breeding pairs (HET × HET) were maintained at **University Health Network (UHN**; Toronto) facilities and replaced every 6 months. All experiments were conducted in compliance with UHN animal use protocols, with oversight from **The Centre for Phenogenomics (TCP**; Toronto), and in accordance with the guidelines of the Canadian Council on Animal Care (CCAC). Animals were housed under standard conditions on a 12-h light/dark cycle (7 am on/7 pm off) with *ad libitum* access to food and water. All experiments were conducted blind to genotype and utilized WT and *Mapt* KO rat or mouse littermate pairs for between-genotype comparisons. Unblinding occurred after completing experiments and data analysis.

Genotyping

Tail or ear notch samples were collected from P8-to-12 rats and mice. Genotyping was performed using an established NaOH extraction protocol (4). Briefly, 75 µl of 25 mM NaOH/0.2 mM EDTA was added to each sample and heated at 95ºC for 15 min. Samples were then centrifuged at 4,000

rpm for 3 min, and 75 µl of 40 mM Tris HCl at pH = 5.5 was added to neutralize the reaction. For polymerase chain reactions (PCR), 1 µl of lysate was mixed with 5 µl of 2 × HS-red Taq mix, 3 µl of ultrapure water, and 1 µl of primers (final concentration: 4 µM). PCR was performed on a T-100 Thermal Cycler that consisted of 35 cycles (in min × ºC): 5 × 95, 0.25 × 95, 0.25 × 65, and 0.5 × 72. Samples were then cooled and maintained on a cycle (in min × ºC): 1 × 72, and ∞ × 12. PCR products were loaded onto a 2 % agarose gel in 1 × tris-acetate-EDTA buffer and subjected to electrophoresis at 180 V for 30 min in a Sub-Cell GT System (Bio-Rad). Gels were imaged using a GelDoc XR+ imaging system.

Immunoblotting

20 µg of protein was loaded onto 7.5% or 10% TGX Stain-Free FastCast polyacrylamide gels, with Precision Plus Protein All Blue Prestained Protein ladder added to one lane. Gels were placed in running buffer (1 × tris-glycine-SDS buffer) and electrophoresed at 180 V until the desired separation was achieved. Total protein levels were tagged and imaged using stain-free technology (Bio-Rad) on a ChemiDoc MP imaging system.

Proteins were transferred onto Immun-Blot 0.45 µm low fluorescence PVDF membranes in transfer buffer (containing: 20% Trans-Blot Turbo 5 × Transfer Buffer, 20% ethanol, and 60% ultrapure water) across two cycles of Turbo-TGX transfer (25 V, 2.5 A, 3 min) on the Trans-Blot Turbo Transfer System. Membranes were rinsed in 1 × tris-buffered saline containing 0.1% Tween 20 (1 × TBST), and total protein levels were imaged. Blocking was performed using Everyblot blocking buffer (Bio-Rad, cat #: 120-100-20) for 30 min. Membranes were incubated overnight (16 h) at 4ºC with a *N*-terminal tau monoclonal antibody (cat #: MABN2406, Millipore Sigma), *C*-terminal tau polyclonal antibody (cat #: A1103, ABclonal) or MAP2 antibody (cat #: MA5-

12826, ThermoFisher Scientific) on a gentle rocker. The following day, primary antibodies were removed, and the membranes were washed 6 times with 1 × TBST before and after incubation with secondary antibodies (StarBright Rabbit-700 (cat #: 12004161) or StarBright Mouse-520 (cat #: 12005866), Bio-Rad) for 1 h at room temperature. Proteins levels were normalized to total stain-free protein levels and expressed as a % relative to male WT rats.

Hippocampal slice preparation and extracellular hippocampal slice electrophysiology

Rodents were anesthetized with isoflurane and decapitated, and the brains were rapidly extracted into chilled **artificial cerebrospinal fluid (ACSF)** consisting of (in mM): 124 NaCl, 10 D-Glucose, 24 $NaHCO_3$, 3 KCl, 1.25 NaH_2PO_4, 1 $MgSO_4$, and 2 $CaCl_2$ saturated with carbogen (95% O_2/5% CO_2). Transverse dorsal hippocampal slices (400 µm thick) were prepared using a vibratome (cat #: VT1200, Leica Biosystems) and recovered for 1–2 h in room temperature ACSF before experiments. ACSF was continuously perfused into the recording chamber at 2.5 mL/min, where the submerged slices were maintained at 29 °C. **Field excitatory postsynaptic potentials (fEPSPs)** were evoked by stimulating the Schaffer collateral-commissural pathway with a bipolar matrix electrode (cat #: MX21XEP(RM1), FHC) and recorded ~300 µm away with a borosilicate glass recording electrode (with a resistance of 1.3–4.0 MΩ). Biphasic constant current stimulus pulses (0.1 ms primary pulse, 0.2 ms half-intensity secondary pulse) were delivered at 0.033 Hz using a stimulus isolator (cat #: STG 4002, Multichannel Systems). LTD was induced by **low-frequency stimulation (LFS**; 900 pulses delivered at 1 Hz) following a 30 min stable baseline, with stimulus intensity set to three times the threshold required to elicit a visually detectable response (~0.1 mV). Responses were recorded for 60 min following the end of LFS. Stimulus intensities ranged from 12-36 µA and did not differ between groups. Signals

were amplified and low pass filtered at 3 kHz (A-M Systems Inc. Model 3000 amplifier) digitised at 40 kHz (National Instruments USB-6341 digitiser), and acquired and analysed using WinLTP (version 2.32, (5)) and Clampfit (version 11.4.3; Molecular Devices) software, respectively.

Data analysis and statistics

All reported *n*-values represent biological replicates. Data are presented as the mean ± standard error of the mean and were analyzed using GraphPad Prism Software (version 11.0.2). For comparison of body, brain and hippocampus weights, and of tau levels with immunoblotting, groups were compared using unpaired, two-tailed Student's *t*-test. For comparing LTD levels, the dataset was checked for homogeneity of variance using Spearman's rank correlation test for heteroscedasticity, and for normality using the D'Agostino–Pearson test prior to two-way ANOVA followed by pairwise comparisons with Šídák correction. The statistical tests used are detailed in the results and/or figure legends and the full numerical values for all statistical tests are in Supplementary Tables 1, 2 and 3.

References

1. Gertsenstein M, Lintott LG, Nutter LMJ. Engineering Base Changes and Epitope-Tagged Alleles in Mice Using Cas9 RNA-Guided Nuclease. Curr Protoc. 2025 Feb;5(2):e70109. doi:10.1002/cpz1.70109 PubMed PMID: 39999224; PubMed Central PMCID: PMC11856344.

2. Filipiak WE, Saunders TL. Advances in transgenic rat production. Transgenic Research. 2006. doi:10.1007/s11248-006-9002-x PubMed PMID: 17009096.

3. Gonçalves RA, Wijesekara N, Fraser PE, De Felice FG. Behavioral Abnormalities in Knockout and Humanized Tau Mice. Frontiers in Endocrinology. 2020. doi:10.3389/fendo.2020.00124

4. Truett GE, Heeger P, Mynatt RL, Truett AA, Walker JA, Warman ML. Preparation of PCR-quality mouse genomic dna with hot sodium hydroxide and tris (HotSHOT). BioTechniques. 2000. doi:10.2144/00291bm09 PubMed PMID: 10907076.

5. Anderson WW, Collingridge GL. Capabilities of the WinLTP data acquisition program extending beyond basic LTP experimental functions. Journal of Neuroscience Methods. 2007. doi:10.1016/j.jneumeth.2006.12.018 PubMed PMID: 17306885.

Supplementary Figures

Supplementary Figure 1. Molecular validation and phenotypic characterisation of *Mapt*^{em1Tcp} rats. (A) Results of Sanger sequencing of the *Mapt* gene (Chromosome 10 base-pairs 92,348,314 to 92,356,727) in *Mapt*$^{-/-}$ (KO) rats showing the sequences upstream (light blue) and downstream (dark blue) of the 7732-bp deleted region and a 3-bp indel at 92,356,440 to 92,356,442. **(B)** Male *Mapt*$^{+/+}$ (WT), *Mapt*$^{+/-}$ (HET), and *Mapt* KO rats were born at expected Mendelian ratios (0.9:2.1:1.0; n = 144 litters). **(C–E)** Masses of (C) body (n = 7 and 7), (D) brain (n = 4 and 4), and (E) both hippocampi (n = 4 and 4) were unchanged between WT and *Mapt* KO rats. All mass comparisons were made using unpaired two-tailed Student's *t*-tests. Detailed statistics are shown in Supplementary Table 3. **(F-I)** Full-length blot images corresponding to the representative images of total tau probed using (F) *N*-terminal monoclonal (MABN2406) and (H) *C*-terminal polyclonal (A1103) antibodies and (G,I) total stain-free protein blots shown in Figure 1B,C. An additional band of unknown identity (denoted by *) was observed in (F) but not (H), likely representing nonspecific antibody reactivity. **(J)** Multiplex of the (left) *C*-terminal polyclonal antibody (A1103) with a (right) monoclonal MAP2 antibody (MA5-12826, ThermoFisher Scientific) revealed cross-reactivity with tau, MAP2C (~75 kDa) and MAP2B (~280 kDa), as evidenced in the (middle) merged image.
