# Supplementary material for: Antithetical roles of tau in hippocampal long-term synaptic depression in rats versus mice
Source: Mol Brain. 2026 Oct 23;19:76. doi: 10.1186/s13041-026-01341-y (PMC13692963; doi:10.1186/s13041-026-01341-y)

A

Chromosome 10: bp# 92348314 to 92356727 of full-length rat 2N4R tau (XM_008768277.3):

**5'...AAGGTTGATGGCCCAGCTGGCACTCTGTGGATGCTTCTGGAAGGCA
ACTTTGCCTCTGGTGTGCCTTGAGGTCAGCATGGGCTGGCTGGCCTTATA
ACCACACACCCCCAACCCGGCTATCCTCGCCCCCAGACTAGTGTGCACG
CCATCCCATTGCTGGGTCCTGATTTCCCCTTGTTCTTGAGATTAGTGCTTA
CCAACTTGGGTCCTGCTCATGTGGTAGGGAACTCCCTCAGCCTTCTTTGT
TTAAGGTGACTGTTAGCTTCTTAAATAGAACCCAAAGACCCCACCCCCAC
CCCAAATCTG—(7732-bp-deletion)—CTGGTCTCATTTGCCAAAAGGCTCT
GCTTCCTGCCTGTTTGCTCTCACTATAACATGTGATGTGTGACCAGAGGA
TGACTGTCACGA(GGC)CATCGCGTTCTATAAAGGCAACGTTTCTGTCTTG
ACACTTAGCACAGGGAGTTGTACTCAGTTCCTCCCCAACGCCTACTTCCT
TCCCTCATAGTTTAAATGAAGGTGTGTGTAAAGTTTGGAGTTTGTACTTCT
GACTCTCACCACTAGAGATCTAGGCTCAGTAAACCTGGAGTGTTTAATAT
ACAGAGCGTACACTGGGAGCCAGAGAGCCAGGATCGAGGGAAGAAGT
GACAAAGGGAAGAAGAGTGAGGGAAGAAGACAAAGGCAGGGAGGATT
CTGGGT...3'**

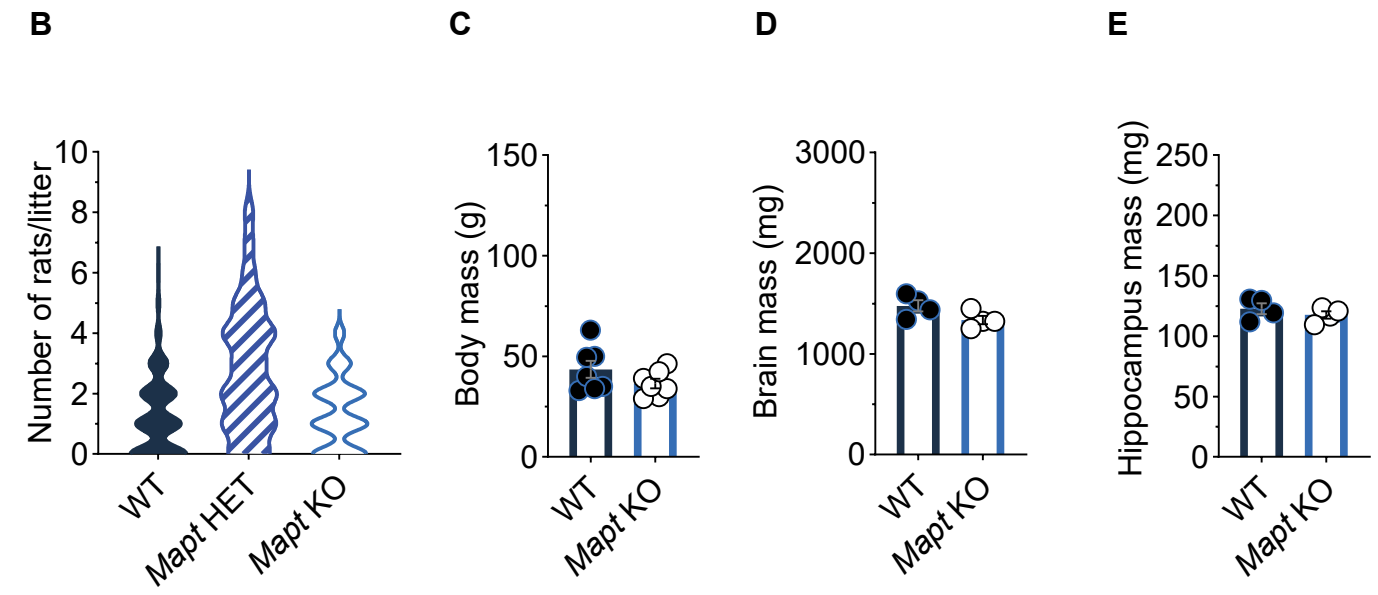

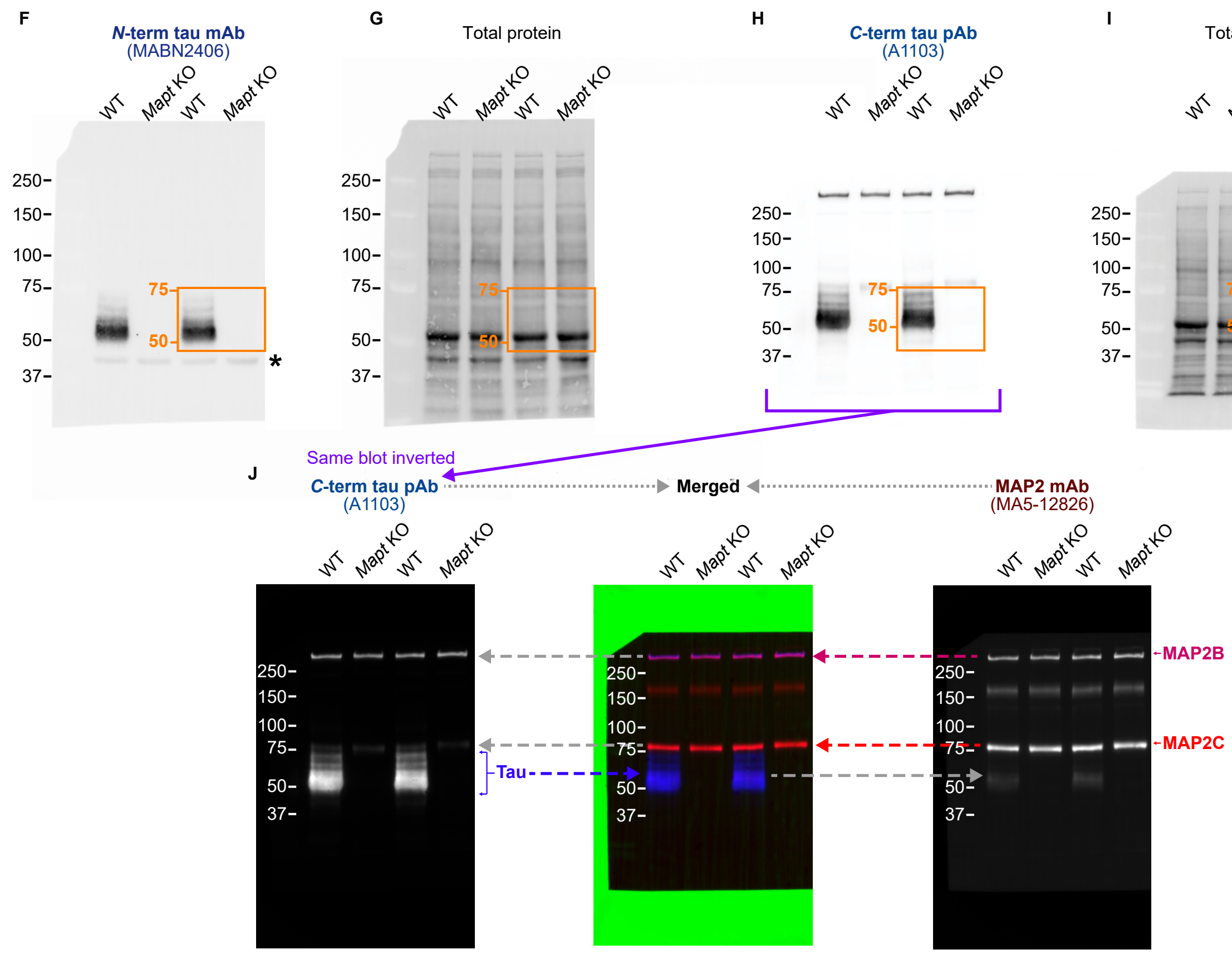

Supplement: Supplementary file 2 [file 13041_2026_1341_MOESM2_ESM.pdf]
