# Supplementary material for: Antithetical roles of tau in hippocampal long-term synaptic depression in rats versus mice
Source: Mol Brain. 2026 Oct 23;19:76. doi: 10.1186/s13041-026-01341-y (PMC13692963; doi:10.1186/s13041-026-01341-y)
Supplement: Supplementary file 3 [file 13041_2026_1341_MOESM3_ESM.docx]

**Supplementary Tables**

| **Group (*n*-value): mean ± s.e.m.:** | **Unpaired *t*-test: *t*-statistic, degrees of freedom, and *P*-values:** |
| --- | --- |
| Total tau (MABN2406; *N*-terminal mAb) levels:  **Male WT (8):** 100.0 ± 2.0%  **Male *Mapt* KO (8):** 0.5 ± 0.1% | *t*_(14)_ = 49.3, *p* < 0.0001 |
| Total tau (A1103, *C*-terminal pAb) levels:  **Male WT (8):** 100.0 ± 15.6%  **Male *Mapt* KO (8):** 0.1 ± 0.02% | *t*_(14)_ = 6.4, *p* < 0.0001 |

**Supplementary Table 1. Summary of *t*-test for immunoblotting experiments.**

| **Group (*n*-value): mean ± s.e.m.:** | **Two-way ANOVA: *F*-statistic, degrees of freedoms (dfn,d), and *P*-values** | **Post-hoc test *t*- and *p*-values:** |
| --- | --- | --- |
| % LTD (1 Hz LFS):  **Mice WT (9):** 29.4 ± 3.8  **Mice *Mapt* KO (9):** 14.9 ± 4.4  **Rats WT (6):** 22.0 ± 6.1  **Rats *Mapt* KO (6):** 40.3 ± 4.4 | **Interaction:**  *F*_(1, 26)_ = 30.5, *p* < 0.0001  **Species effect:**  *F*_(1, 26)_ = 9.2, *p* = 0.006  **Genotype effect:**  *F*_(1, 26)_ = 0.4, *p* = 0.5 | **Mice: WT vs. *Mapt* KO:**  *t*_(26)_ = 3.9, *p* = 0.003  **Rats: WT vs. *Mapt* KO:**  *t*_(26)_ = 4.0, *p* = 0.002  **WT: Mice vs. Rats:**  *t*_(26)_ = 1.8, *p* = 0.3  ***Mapt* KO: Mice vs. Rats:**  *t*_(26)_ = 6.0, *p* < 0.0001 |

**Supplementary Table 2. Summary of two-way ANOVA and post hoc pairwise comparisons for LTD experiments.**

| **Group (*n*-value): mean ± s.e.m.:** | **Unpaired *t*-test: *t*-statistic, degrees of freedom, and *P*-values:** |
| --- | --- |
| Body mass (g): **WT (7):** 43.5 ± 6.4  ***Mapt* KO (7):** 36.5 ± 3.7 | *t*_(12)_ = 1.4, *p* = 0.2 |
| Brain mass (mg)**:  WT (4):** 1476.8 ± 55.1  ***Mapt* KO (4):** 1336.8 ± 41.7 | *t*_(6)_ = 2.0, *p* = 0.1 |
| Hippocampus mass (mg):  **WT (4):** 122.9 ± 5.2  ***Mapt* KO (4):** 117.6 ± 3.5 | *t*_(6)_ = 1.0, *p* = 0.4 |

**Supplementary Table 3. Summary of *t*-test for body, brain and hippocampal mass comparisons.**
